# CAR-iNKT cells: redefining the frontiers of cellular immunotherapy

**DOI:** 10.3389/fimmu.2025.1625426

**Published:** 2025-07-11

**Authors:** Magdalena Niedzielska, Amy Chalmers, Martyna C. Popis, Efrat Altman-Sharoni, Stephen Addis, Rebekka Beulen, Nils-Petter Rudqvist, Eleni Chantzoura, Marco A. Purbhoo, Dhan Chand, Mark A. Exley

**Affiliations:** ^1^ Research & Development, MiNK Therapeutics Inc., Lexington, MA, United States; ^2^ Research & Development, Agenus Inc., Lexington, MA, United States; ^3^ Gastroenterology, Brigham & Women’s Hospital, Boston, MA, United States; ^4^ Life Sciences, Imperial College, London, United Kingdom

**Keywords:** iNKT cells, CD1d, cancer immunotherapy, CAR, adoptive cell therapy (ACT)

## Abstract

Despite significant advances in cancer therapies, many malignancies remain resistant to current treatments due to complex immunosuppressive mechanisms, limited neoantigen expression, and dynamic tumor adaptations, underscoring the need for innovative therapeutic strategies. Adoptive cell therapy (ACT), particularly with chimeric antigen receptors (CARs and recombinant TCRs) targeting cancer-associated antigens, has emerged as a transformative strategy. However, conventional CAR-T cell therapies face substantial limitations such as manufacturing challenges, severe toxicities, and limited efficacy against solid tumors. Invariant natural killer T (iNKT) cells, a unique lymphocyte subset bridging innate and adaptive immunity, have emerged as a compelling alternative platform for CAR-based therapies, due to their distinctive ability to persist, penetrate in and remodel the tumor microenvironment (TME). Unlike conventional T cells, iNKT cells exhibit rapid activation without priming, potent cytotoxicity, and extensive immunomodulatory functions. Furthermore, the inherent immunomodulatory properties of iNKT cells through interactions with the monomorphic antigen-presenting molecule CD1d or stress ligands augment endogenous anti-tumor immunity by activating NK cells and cytotoxic T lymphocytes, promoting dendritic cell maturation, and reducing immunosuppressive myeloid cells, unlike other Innate T cells. CAR-engineered iNKT (CAR-iNKT) cells therefore leverage multiple targeting mechanisms through their native semi-invariant T-cell receptor (TCR), NK receptors (NKRs) and engineered CARs, enabling broader and more effective tumor recognition while actively reshaping immunosuppressive TME. Notably, iNKT cells lack alloreactivity, circumventing the risk of graft-versus-host disease (GvHD), positioning CAR-iNKT cells as ideal candidates for “off-the-shelf” allogeneic therapies that can overcome the limitations of existing immunotherapies.

## Introduction

Cancer remains a major global health and socioeconomic challenge, with steadily rising incidence rates, particularly among adults under 50, and an expected 77% increase in new cases by 2050 (1, 2), underscoring the urgent need for innovative therapeutic strategies. A revolution in understanding cancer and immunotherapy over the past decade has fundamentally transformed treatment paradigms by exploiting the immune system’s intrinsic ability to recognize and eliminate malignant cells (3).

Adoptive cell therapy (ACT) using chimeric antigen receptor (CAR) technologies, antibody-like membrane bound antigen receptors which redirect immune cells toward specific tumour antigens with high precision, represents one of the most significant breakthroughs in immunotherapy. Notably, autologous CAR-T cells have demonstrated exceptional efficacy in hematological malignancies, resulting in multiple FDA approvals for products targeting CD19 and B-cell maturation antigen (BCMA) (4, 5). In some patients, these “living drugs” persist for many years, functioning as long-term sentinels that sustain remission and further underscore the curative potential of engineered cellular immunotherapy (6).

Despite these impressive achievements, CAR-T cell efficacy against solid tumors remains limited to date, primarily restricted by a highly immunosuppressive TME that impairs T cell trafficking and infiltration into tumor sites, hostile metabolic challenges, tumor antigen heterogeneity and escape (7–9). Moreover, life-threatening toxicities, including cytokine release syndrome (CRS) and immune effector cell-associated neurotoxicity syndrome (ICANS) (10, 11), coupled with prohibitive manufacturing and treatment costs that limit accessibility (12, 13), remain significant clinical issues and limit eligibility. To overcome these barriers, researchers are reimagining CAR therapy by engineering autologous and allogeneic (healthy donor) non-conventional immune effector cells with improved tumor-homing capacity, persistence, intrinsic TME-modulating activity, cytotoxicity and in the case of allogeneic cells, reduced alloreactivity for universal use.

Invariant Natural Killer T (iNKT) cells, a subset of T lymphocytes with innate-like activity have emerged as promising candidates for next-generation CAR-based therapies. These unique lymphocytes are defined by their semi-invariant T cell receptor (TCR), which recognizes lipid antigens presented by CD1d, a conserved non-polymorphic MHC class I-like molecule (14, 15). This distinct recognition mechanism enables iNKT cells to bypass traditional HLA restrictions, a critical feature for developing truly “off-the-shelf” allogeneic therapies with broader patient reach than traditional CAR-T cells. Functionally, iNKT cells offer several advantages over conventional T cells for cancer immunotherapy. Coupling TCR specificity with innate-like speed, iNKT cells are rapidly activated without requiring priming and demonstrate potent cytotoxicity (via perforin, granzyme B and FasL/CD95) against various tumor types. Moreover, iNKT cells secrete high levels of multiple cytokines and actively remodel the TME towards a proinflammatory anti-tumor phenotype by removing key barriers such as tumor associated macrophages (TAMs) and myeloid-derived suppressor cells (MDSC). iNKT cells also enhance broader immune surveillance via activation/maturation of dendritic cells and lymphocytes, including NK cells and cytotoxic T lymphocytes (16–19). Given these unique attributes, engineered iNKT cells (e.g., CAR-iNKT, TCR-iNKT) are being developed to further optimize tumor killing and extend the reach of cellular immunotherapy to immunologically ‘cold’ and difficult-to-treat solid tumors (20–22).

In this review, we examine the unique biological properties of iNKT cells, recent advances in CAR-iNKT and TCR-iNKT engineering, emerging clinical data, and strategies to overcome current challenges, providing a comprehensive assessment of how this innovative platform is redefining cellular cancer immunotherapy.

## iNKT cell identity and functional heterogeneity

CD1d-restricted Natural Killer T (NKT) cells represent a heterogenous T cell subset characterized by their ability to recognize lipid antigens presented by the MHC class I-like molecule, CD1d. Among NKT cells, iNKT cells, also known as type I NKT cells, are the best-characterized subpopulation with relative homogeneity but functional heterogeneity. This subset of unconventional αβ T lymphocytes are defined by their semi-invariant TCR, comprising a near-germline TCRα chain (specifically Vα24-Jα18 in humans and Vα14-Jα18 in mice) paired with a limited TCRβ chains (predominantly Vβ11 in humans and Vβ8, Vβ7, or Vβ2 in mice). Additionally, iNKT cells express several NK cell-associated surface molecules, including CD161 or NKG2D (18, 23) reflecting their hybrid phenotype between innate and adaptive lymphocyte.

The recognition of lipid antigens by iNKT cells occurs via binding to CD1d, a monomorphic antigen-presenting molecule predominantly expressed on professional antigen-presenting cells (APCs), such as dendritic cells and macrophages, as well as B cells. Alpha-galactosylceramide (α-GalCer), originally isolated from marine sponges, is considered the prototypical and potent lipid antigen recognized by iNKT cells (24). However, α-GalCer and structurally related lipids are also produced by bacteria including gut microbiota (25), and have recently been identified as endogenous mammalian antigens for iNKT cells (26). Beyond α-GalCer, additional endogenous iNKT cell ligands include phospholipids and glycosphingolipids, such as glucosylceramide (27). A distinctive feature of iNKT is their unique developmental programming. Unlike conventional T cells that require priming, iNKT exit the thymus as fully functional effectors, capable of rapid cytokine production and cytotoxic responses (18, 28). As such, iNKT can play pivotal roles in modulating immune responses during infections and in tumor immune surveillance, partly by promoting DC maturation and activating NK cells as well as CD8^+^ T lymphocytes (18).

Despite their common TCR architecture and therefore specificity, iNKT cells exhibit substantial phenotypic and functional diversity. iNKT cells are rare cells and need precise detection reagents, including a monoclonal antibody (6B11), that recognizes primate iTCR rearrangements (29) and lipid antigen-loaded CD1d-multimers (30). Human iNKT can be classified based on CD4 and CD8 expression into four main subsets: CD4^+^CD8^-^ (CD4^+^), CD4^-^CD8^+^ (CD8^+^), CD4^-^CD8^-^ (double negative; DN), and CD4^+^CD8^+^ (double positive; DP, mainly thymic early iNKT) (29, 30). These subsets exhibit distinct tissue homing properties through differential chemokine receptor expression. One of 2 major peripheral subsets, CD4^+^ iNKT cells predominantly express CCR4, a chemokine receptor associated with homing to (e.g.) the lung (18, 31–33). In contrast, the rarer CD8^+^ and other major subset, DN iNKT preferentially express CCR1, CCR6, and CXCR6, with the latter being associated with iNKT homing, retention and survival within hepatic and pulmonary tissues (23, 30).

Functionally, iNKT cell subsets parallel the classical T helper (Th) cell lineages. Th1-like iNKT, predominantly found within the DN and CD8 subsets, express the transcription factor T-bet, secrete IFN-γ and TNF-α upon activation, express high levels of the activating receptor NKG2D, and exhibit potent cytotoxicity (23, 30). Mixed Th1-Th2-like iNKT, primarily CD4^+^, express GATA-3, produce IL-4, IL-13, and IFN-γ, and support humoral immunity (23, 34). Th17-like iNKT express RORγt and secrete IL-17, IL-21, and IL-22 and promote mucosal immunity and inflammation. Additional functional subsets include Treg-like iNKT that express FOXP3 and secrete IL-10, and T follicular helper (Tfh)-like iNKT, which produce IL-21 and support B cell function (23, 35).

A defining characteristic of iNKT cells is their remarkable functional plasticity. Their cytokine production profiles adapt in response to specific microenvironmental cues and immunological contexts. This functional flexibility allows iNKT to respond dynamically to diverse pathological conditions, highlighting their pivotal role in bridging innate and adaptive immunity (23). Such adaptability is potentially valuable in cancer, where iNKT can recalibrate their effector functions in response to the complex and evolving tumor microenvironment, as well as other immunotherapies.

## From entry to eradication: how iNKT cells infiltrate tumors and drive anti-tumor immunity

Effective tumor infiltration by immune cells is critical for shaping the tumor microenvironment and can significantly influence disease progression and response to immunotherapy. Emerging evidence demonstrates that iNKT cells possess remarkable capabilities to infiltrate tumor tissues and orchestrate multi-faceted anti-tumor responses through both direct and indirect mechanisms (19).

For example, in MC38 colorectal tumor models using *Vα14 Tg Cxcr6^Gfp* mice, iNKT preferentially migrate to sites of disease, rather than normal colon tissue (36). These tumor-infiltrating iNKT cells exhibit an activated phenotype, characterized by CD69 and FasL expression, and production of effector molecules such as IFN-γ and Granzyme B (36, 37). Similarly, in the TRAMP prostate cancer model, iNKT are recruited to the TME via the CCL2-CCR5 chemokine axis (38). Notably, human prostate tumor and epithelial cells can express CD1d, enabling direct interaction with iNKT cells (38, 39). However, like conventional T cells, intratumoral iNKT often accumulate at the tumor periphery, suggesting that physical and immunological barriers restrict deeper infiltration into tumor cores. These barriers are largely mediated by interactions with CD1d-expressing TAM. Strategies aimed at enhancing iNKT mobility and modulating these interactions represent promising approaches to overcome these limitations (37).

**Figure 1:**
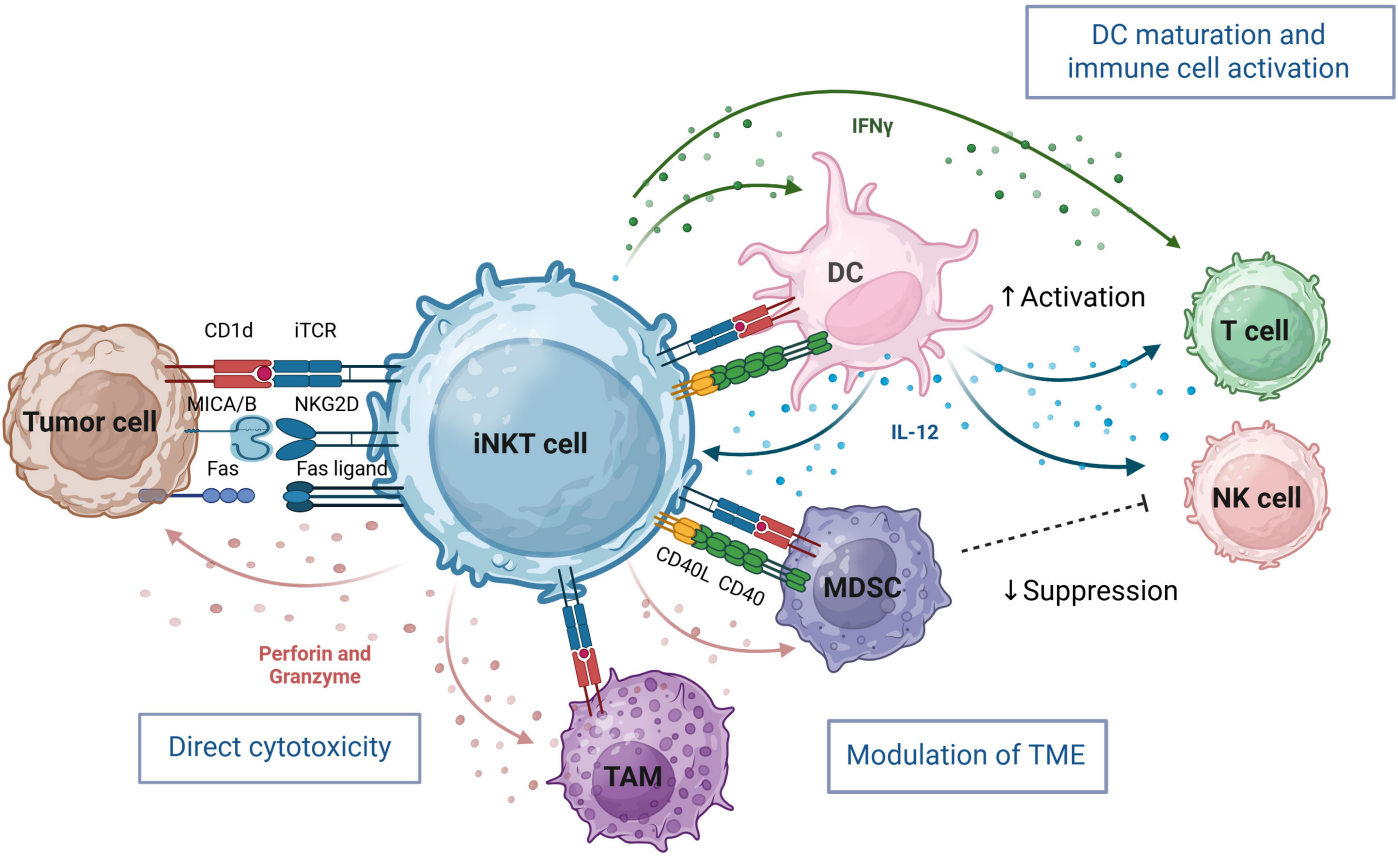
iNKT cells bridge innate and adaptive immunity through cytotoxic activity and immune-enhancing adjuvant effects. iNKT cells orchestrate potent anti-tumor responses through direct cytotoxicity, immune cell cross-talk, and tumor microenvironment (TME) remodeling. They directly kill tumor cells via invariant iTCR recognition of CD1d–lipid complexes and through NK cell receptors such as NKG2D which recognize stress ligands on tumor cells. iNKT cells also target immunosuppressive tumor-associated macrophages (TAM) and Myeloid-derived suppressor cells (MDSC), creating a more permissive environment for NK and T cell infiltration and function. Indirectly, iNKT-derived IFN-γ activates NK cells, promotes dendritic cell (DC) maturation, and enhances T cell responses. Bidirectional activation with DCs—mediated by CD1d, CD40–CD40L, and IL-12 further amplifies iNKT effector functions. iNKT cells enlarged for clarity, not to scale relative to others. Image created in BioRender.

Within the tumor, iNKT cells can mount a multi-pronged attack to recondition the TME and enhance anti-tumor immunity (**Figure 1**). They can directly lyse CD1d-positive malignancies, including hematologic and select solid tumors, and stress ligand-expressing tumor cells via Fas/FasL interactions, TRAIL-mediated apoptosis, or release of cytotoxic granules containing perforin and granzymes (23, 39, 40). Beyond their direct cytotoxic functions, iNKT serve as powerful orchestrators of broader anti-tumor immune responses. Activation through the TCR leads to upregulation of IL-12 receptors and CD40L, which in turn promotes DC maturation. This bidirectional interaction creates a positive feedback loop that amplifies IFNγ secretion, upregulates MHC class I and II antigen presentation machinery, and promotes Th1 polarized immune responses (18, 39, 41, 42). Furthermore, the activation cascade initiated by iNKT extends to multiple immune compartments, leading to enhanced NK cell activation, and potentiation of B cell and cytotoxic T cell responses, effectively bridging innate and adaptive immunity to generate a coordinated, multi-faceted anti-tumor response.

Notably, iNKT cells also directly target immunosuppressive components within the TME. In preclinical neuroblastoma models, iNKT eliminate CD1d-expressing TAM and inhibit the function of myeloid-derived suppressor cells (MDSC) in a CD1d- and CD40-dependent manner, thereby creating a more favorable microenvironment for effector immune responses (23, 43, 44). This combination of direct cytotoxicity, immunomodulation and TME reprogramming positions iNKT as promising candidates for next-generation cellular engineered immunotherapies.

## Clinical snapshot: harnessing unmodified iNKT cells for immunotherapy

Insights from preclinical murine models to early clinical studies have provided compelling evidence for the intrinsic anti-tumor capacity of iNKT. Retrospective analyses across a broad spectrum of malignancies, including colorectal and hepatocellular carcinoma, acute myeloid leukemia, head-and-neck squamous cell carcinoma, neuroblastoma, upper-gastrointestinal cancers, prostate and lung cancers, show that elevated intratumoral or peripheral iNKT frequencies correlate with improved overall survival (45). These observations provided a strong rationale for therapeutic approaches designed to enhance iNKT cell frequency and function in cancer patients.

Initial clinical trials focused on adoptively transferred, ex vivo expanded autologous and later allogeneic iNKT cells in patients with non-small cell lung cancer, head and neck squamous cell carcinoma, hepatocellular carcinoma, pancreatic cancer and advanced melanoma (**Table 1**). These early-phase studies demonstrated encouraging clinical benefits and a favorable safety profile, with only five treatment-related adverse events of grade ≥2 reported among many patients treated (46–53), underscoring the exceptional tolerability of this approach compared to other cellular immunotherapies.

**Table 1:** I Summary of iNKT cell-based clinical trials for adoptive cellular therapy of cancer.

Trial ID	Status	Study design	iNKT cell product	Indication	Intervention/Dosing	Response/Toxicity	Reference
**-**	Completed	Phase 1, open-label, single arm, dose-escalation study	Autologous iNKT cells enriched bulk PBMCs. Ex vivo expansion with α-GalCer-pulsed PBMCs + IL-2.	Advanced or recurrent non-small cell lung cancer (NSCLC)	Activated iNKT cells administered intravenously. Dose 1: 1x10^7^ cells/m^2^ per injection Does 2: 5x10^7^ cells/m^2^ per injection	SD (4/6). PD (2/6) No major (grade >2) toxicity or severe side effects observed.	(46)
UMIN000000722	Completed	Phase 1, open-label, single-arm, single dose study.	Autologous iNKT cells enriched bulk PBMCs. Ex vivo expansion with α-GalCer-pulsed APC + IL-2.	Recurrent/Refractory head and neck squamous cell carcinoma	1x10^8^ α-GalCer‐pulsed APC administered into nasal submucosa on day 7 and 13 followed by trans‐catheter arterial infusion of activated 5x10^7^ iNKT cells into a tumor‐feeding artery on day 14.	PR (3/8), SD (4/8), PD (1/8). ORR of 38%. One grade 3 AE associated with a PR (fistula formed by collapsing tumor).	(47)
UMIN000000852	Completed	Phase 2, open-label, single-arm single dose study.	Autologous iNKT cells enriched bulk PBMCs. Ex vivo expansion with α-GalCer-pulsed APC + IL-2.	Recurrent head and neck squamous cell carcinoma	1x10^8^ α-GalCer-pulsed APCs administered nasally on day 7 followed by intra-arterial infusion of activated 5x10^7^ iNKT cells via tumor-feeding arteries on day 14.	PR (5/10), SD (5/10). ORR of 50%. No major (grade >2) toxicity or severe side effects observed.	(48)
NCT00631072	Completed	Phase 1 open-label, single-arm, multiple dose study.	Autologous, 6B11-purified iNKT cells. Ex vivo expanded with anti-CD3, irradiated PBMC + IL-2.	Advanced stage Melanoma (III-IV)	Pre-administration of GM-CSF in 6/9 patients. iNKT cells administered in 3 equal doses (up to 2.2x10^8^) by intravenous infusion on days 1, 15 and 29.	NED/SD (6/9), PD (3/9). 67% ORR. No major (grade >2) toxicity or severe side effects observed.	(49)
NCT03175679	Completed	Phase 1 open-label, dose‐escalation study.	Autologous, purified iNKT cells. Ex vivo expanded with α-GalCer-pulsed PBMC + IL-2 followed by co-culture with APCs.	Hepatocellular CARcinoma	iNKT cells administered intravenously in conjunction with IL-2 and along with lymphodepleting chemotherapy (Tegafur) Dose 1: 3x10^7^/m2 Dose 2: 6x10^7^/m2 Dose 3: 9x10^7^/m2 For each patient, 10%, 30%, and 60% of the total dose of iNKT cells were administered over 3 consecutive days.	Four patients were progression-free at 5.5, 6-, 7-, and 11-months post-therapy, and one patient was alive and without tumor recurrence at the last follow-up. Three grade 3 adverse events reported. No DLT.	(50)
NCT05962450	Recruiting	Phase 2 open-label, randomized, controlled, clinical trial.	Autologous iNKT.	Hepatocellular carcinoma	iNKT cells administered intravenously in conjunction with anti-PD1 and Regorafenib. iNKT cells infused i.v. every two weeks as a course of treatment for up to six courses, reinfusion dose is determined according to the patient’s body surface area, about 10^8^~10^9^ cells/m^2^.	No results reported	
NCT04011033	Completed	Phase 2 open-label, randomized, multicenter clinical trial.	Autologous, purified iNKT cells. Ex vivo expanded with α-GalCer-pulsed PBMC + IL-2 followed by co-culture with APCs.	Hepatocellular carcinoma	5×10^8^-10^9^/m^2^ iNKT cells infused at 1st, 3rd, 5th, 7th, 9th, 11th week after first TAE/TACE therapy.	PFS 3 months longer in TAE-iNKT group compared with TAE group. OS 8 month longer. ORR of 51,9%, DCR of 85,2%. Grade 3 adverse events were reported in 1 of 27 TAE-iNKT group patients.	(51)
NCT03551795	Completed	Phase 1/2 clinical trial.	Autologous iNKT cells.	Malignant solid tumors with tuberculosis	Two infusions of iNKT cells (108, 109) in one course of treatment.	No results reported	
NCT03198923	Unknown status	Phase 1/2	Autologous iNKT cells.	Non-small cell lung cancer	Patients are infused with 10 doses of (2-2.5)x10^9^ NK and NKT cells in one course of treatment.	No results reported	
NCT03093688	Unknown status	Phase 1/2 single-arm, single-center clinical trial.	Autologous iNKT cells enriched bulk PBMCs. Ex vivo expansion with α-GalCer-pulsed APC + IL-2, IL-7, IL-15 and IL-12.	Advanced pancreatic cancer	Combined immunotherapy of iNKT and PD-1^+^CD8^+^ T. Autologous iNKT-enriched cells (2x10^8^ to 3x10^9^) were intravenously infused on the first day and autologous iNKT cells (2x10^8^ to 3 x10^9^) mixed with PD-1^+^CD8^+^ T cells (2x10^7^ to 2x10^9^) were transfused on the third day during each course. 9 patients received at least three cycles of treatment.	2 PR. The OS time of 6 patients has been prolonged beyond 12 months. The 1-year survival rate was 66.7%. No major (grade >2) toxicity or severe side effects observed.	(52)
NCT03093688	Unknown status	Phase 1/2 single-arm, single-center clinical trial.	Autologous iNKT cells enriched bulk PBMCs. Ex vivo expansion with α-GalCer-pulsed APC + IL-2, IL-7, IL-15 and IL-12.	Advanced or metastatic NSCLC	Autologous DCs (5x10^5^) mixed with iNKT cells (1x10^8^–1x10^10^) were intravenously transfused on the first day, and autologous DCs (5x10^5^) mixed with iNKT cells (1x10^8^–1x10^10^) and CD8 + PD-1+ T cells (1x10^7^–1x10^9^) were transfused on the third and fourth day during every course. All of them received at least 4 cycles of treatment.	SD (3/3). OS > 5 months. No treatment-related SAEs or grade 3–4 AE observed.	(53)
NCT02619058	Unknown status	Phase 1, open label, dose escalation clinical trial.	Autologous iNKT cells.	Advanced stage Melanoma (III-IV)	Iintravenous administration of autologous NKT cells. NKT cells single low dose: 1x10^9^ on d1, 2x10^9^ on d3, 4x10^9^ on d29, 8x10^9^ on d31. NKT cells single high dose: 5x10^9^ on d1, 5x10^9^ on d3, 5x10^9^ on d29, 5x10^9^ on d31. NKT cells multiple dose: 5x10^9^ on d1, 5x10^9^ on d3 of each 28 days-cycle, the dosing will be ended after 8 cycles.	No results reported	
NCT02562963	Unknown status	Phase 1/2 clinical trial.	Autologous iNKT cells	Advanced non-small cell lung cancer, advanced gastric cancer, advanced hepatocellular carcinoma, advanced colorectal cancer	Patients are infused with two doses of (4 ± 0.5)x10^9^ iNKT cells in one course of treatment.	No results reported	
NCT01801852	Unknown status	Phase 1 clinical trial.	Autologous iNKT cells	Metastatic, treatment-refractory breast cancer, glioma, hepatocellular carcinoma, squamous cell lung cancer, pancreatic cancer, colon cancer or prostate cancer.	iNKT cells treatment plus regular treatment.	No results reported	
NCT04754100	Completed	Phase 1, open-label, dose escalation clinical study.	Allogeneic iNKT cells isolated and ex vivo expanded with α-GalCer-pulsed irradiated PBMCs (agenT-797).	Relapsed/refractory multiple myeloma	agent-797 cells administered intravenously. Dose -1: 4.3x10^5^ cells/kg Dose 1: 1.4x10^6^ cells/kg Dose 2: 4.3x10^6^ cells/kg Dose 3: 1.4x10^7^ cells/kg	No DLTs or related adverse events reported.	(154)
NCT05108623	Completed	Phase 1, open-label, dose escalation clinical study.	Allogeneic iNKT cells isolated and ex vivo expanded with α-GalCer-pulsed irradiated PBMCs (agenT-797).	Relapsed/refractory solid tumors	Monotherapy or in combo with pembrolizumab and nivolumab (anti-PD1). agent-797 cells administered intravenously. Dose -1: 1.4x 10^6^ cells/kg Dose 1: 4.3x10^6^ cells/kg Dose 2: 1.4 x10^7^ cells/kg	1 PR, 2 SD. No grade ≥ 3 neurotoxicity or CRS were observed. No DLTs.	(56)
NCT06251973	Recruiting	Phase 2, open-label, single-arm clinical study.	Allogeneic iNKT cells isolated and ex vivo expanded with α-GalCer-pulsed irradiated PBMCs (agenT-797).	Metastatic or advanced unresectable adenocarcinoma of esophageal, gastric, or gastroesophageal junction	Combination of agenT-797, botensilimab (Fc-enhanced anti-CTLA-4), and balstilimab (anti-PD-1), with ramucirumab and paclitaxel. Single infusion of agenT-797 at a dose of 1.4x10^7^ cells/kg.	No results reported	(57)

The table summarizes previous and ongoing iNKT cell clinical trials and latest data, detailing iNKT cell product, study design, indication and response/toxicity. References are provided when available. ORR, overall response rate; PR, partial response; CR, complete response; SD, stable disease; PD, progressive disease; NED; no evidence of disease; SAE; serious adverse event; AE, adverse event; PFS, progression free survival; OS, overall survival; DCR, disease control rate; DLT, dose‐limiting toxicity; SAE, serious adverse event; iNKT,; IFN-γ, Interferon-γ, TTR, time to response; α-GalCer, α-Galactosylceramide; PBMC, peripheral blood mononuclear cell; IL-2, interleukin 2; APC, antigen presenting cells; PD-1, programmed cell death protein 1; TAE, transarterial embolization; TACE, transarterial chemoembolization; Fc, fragment crystallizable.

Unlike conventional T cells, which pose significant risks when used across HLA barriers, iNKT cells can be safely transferred between unmatched donors and recipients without causing GvHD. Their restricted TCR recognizes the non-polymorphic CD1d molecule rather than variable HLA complexes, enabling their use as universal donor cells. Recently, an allogeneic iNKT therapy, agenT-797 (MiNK Therapeutics), has entered clinical development as an off-the-shelf immunotherapy, comprising of over 99.5% unmodified human iNKT cells isolated from healthy donors mononuclear cell apheresis and vastly expanded ex vivo to treat many recipients per donor. To accumulate as much as practical of this allogeneic product, where quality and quantity are more important than speed, expansions take about 6 weeks with simply low dose IL-2. AgenT-797 functions as a potent immune modulator with clinical proof-of-concept demonstrated across multiple disease indications, including acute respiratory distress syndrome (ARDS), hematologic and solid tumors (54–56). Notably, among 82 treated patients, there were no occurrences of severe CRS, GvHD, or ICANS events (54, 56, 57), reflecting the non−alloreactive nature of iNKT cells. Interestingly, there was also no evidence of depletion of CD1d+ monocytes, B cells or other cells that iNKT interact with, presumably since no high avidity exogenous antigen was simultaneously administered (54, 56, 57).

In a heavily pre−treated solid−tumor cohort (N=34), agenT-797 demonstrated meaningful clinical activity, including partial responses in third-line gastric cancer and testicular cancer, and prolonged disease stabilization in nine additional patients (55, 56). In the gastric cancer cohort, paired tumor biopsies revealed a transformation from ‘cold’ immune deserts to inflamed microenvironments following agenT-797 therapy, characterized by enhanced infiltration of cytotoxic T cells and dendritic cells, and activation of both central and effector memory T cells (55, 58). Additionally, peripheral blood analyses demonstrated elevated levels of pro-inflammatory cytokines, including IFN-γ post-treatment (55, 58). While direct detection of infused iNKTs within tumor tissue remains limited, post-treatment biopsies showed robust immune remodeling, consistent with their functional activity in the tumor and active engagement of innate immune cascades (55, 58). Moreover, mechanistic studies using clinically relevant human models demonstrated that agenT-797 rescues exhausted T cells, activates dendritic cells, and selectively targets immunosuppressive M2 macrophages while sparing M1 macrophages, providing a mechanistic basis for agenT-797’s ability to reshape the TME toward the more immunostimulatory state observed in post-treatment clinical specimens (54, 55, 58).

Collectively, these clinical findings underscore the transformative potential of iNKT cell-based therapies in cancer treatment. Their unique ability to directly kill tumor cells, reprogram the TME and activate multiple immune effector mechanisms with favorable safety profiles positions them as a promising approach and platform to overcome critical barriers associated with current cellular immunotherapies and expanded therapeutic potential across a broader spectrum of malignancies.

## CAR design and optimization for iNKT cell-based immunotherapy

Adding a CAR to iNKT cells enhances their tumor-targeting specificity and cytotoxicity, combining the innate-like, tissue-homing and immunoregulatory properties of iNKT cells with the precision of CAR-mediated antigen recognition for improved anti-tumor efficacy. CARs were originally designed for conventional T cells, and the majority of work has been done with this cell type. With the emergence of novel cellular platforms, such as iNKT cells, CARs are increasingly being adapted and integrated into diverse immune cell types due to their standardized design and proven efficacy.

**Figure 2:**
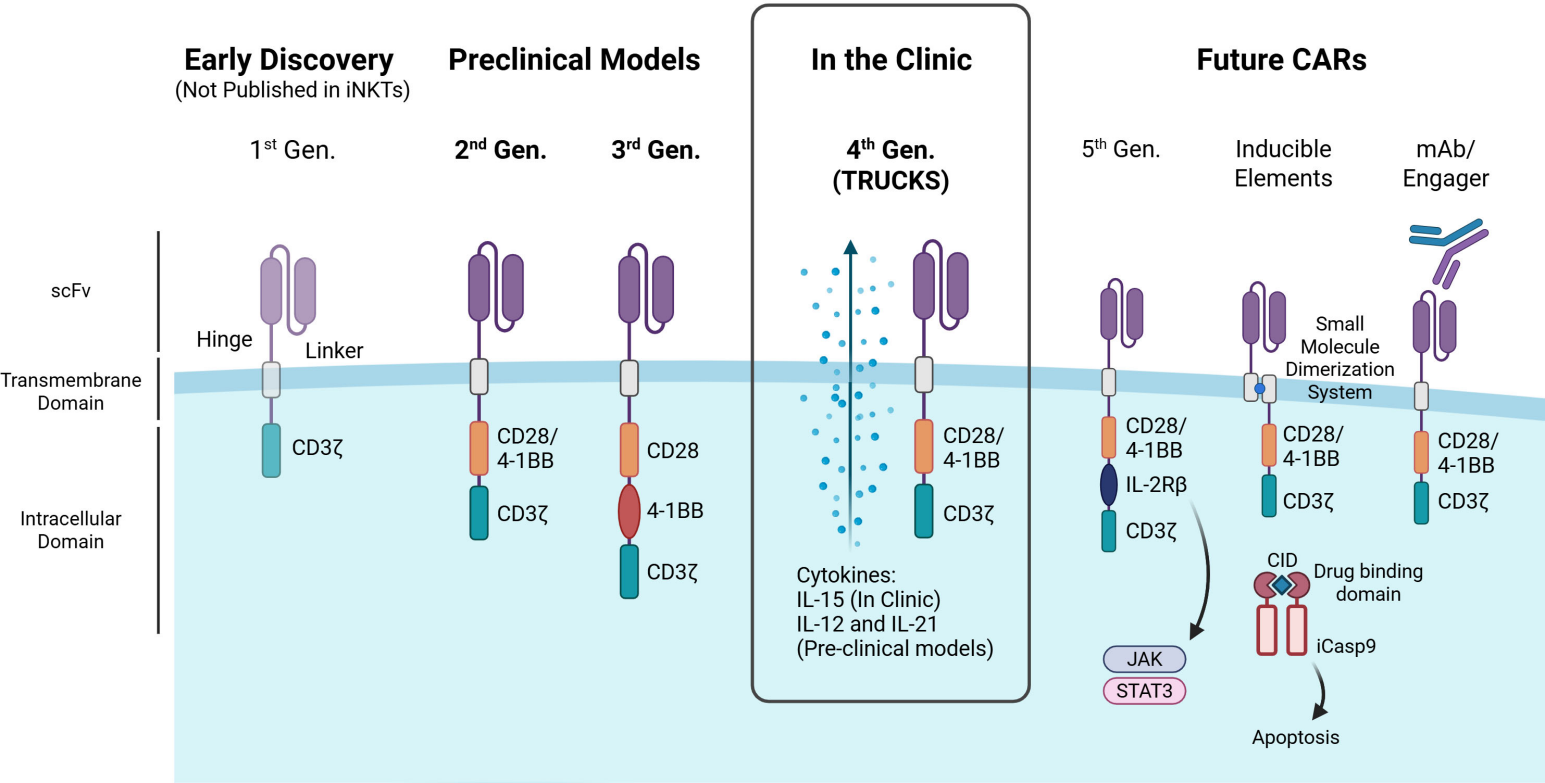
Advances in CAR design and their application in iNKT cells. The figure illustrates the evolution of chimeric antigen receptor (CAR) generations, highlighting key structural components and advancements. First-generation CARs contain a single CD3ζ signaling domain, but have not been reported in iNKTs. Second-generation CARs enhance T cell activation with a costimulatory domain (e.g., CD28, 4-1BB), while third-generation CARs integrate multiple domains for improved function. Both second- and third-generation CARs have been tested in iNKT preclinical models. Fourth-generation CARs (aka ‘TRUCKs’) have been used in iNKT clinical trials and introduce cytokine expression for better iNKT persistence, cytotoxicity and/or expansion. Promising future CAR designs for iNKT cells include: fifth-generation CARs with cytokine receptor domains (e.g., IL-2Rβ) linked to JAK-STAT signaling to boost proliferation, persistence, and anti-tumor activity; CARs with inducible safety features such as small molecule dimerization systems or kill switches (e.g., iCasp9); and CARs that target tumor cells via monoclonal antibodies or engager molecules. CID – Chemical Inducer of Dimerization. Image created in BioRender.

CARs are made up of 4 main components: the antigen binding domain displayed extracellularly, a spacer/hinge region, a transmembrane domain (TMD) and one or more intracellular domains (ICD) which mediate signal transduction (**Figure 2**) (59, 60). The antigen binding domain recognizes the given antigen and is most commonly a single-chain variable fragment (scFv) due to their high specificity and affinity. scFv’s are derived from the variable heavy and light chains of monoclonal antibodies connected by a flexible peptide linker. When selecting a scFv for use in CAR T-cell therapies, it’s crucial to carefully consider its affinity. The scFv must bind strongly enough to trigger a significant activation response, but not so strongly that it causes excessive stimulation. Unlike monoclonal antibodies, CARs with too high an affinity can lead to detrimental effects, such as activation-induced cell death, T cell exhaustion, or toxicity due to CRS. Balancing the affinity is key to achieving effective immune responses while minimizing these risks (61–63). The optimal scFv for CARs success must also consider scFv epitope location, density of the target antigen and ligand-independent tonic signaling (64, 65). The flexibility and length of the linker between the variable heavy and light chains can also significantly influence CAR function by affecting the stability and proper folding of the scFv (66).

The scFv is attached to the transmembrane domain via the spacer (hinge) component which is commonly derived from amino acid sequences found in CD8, CD28, IgG1 or IgG4 (67). Although the hinge region has been less researched than the antigen binding domain or ICDs, studies indicate altering the length or flexibility of the hinge region cause significant effects on CAR-T cytotoxicity (68–70). Zhang et al., indicated by removing 2 consecutive glycine residues in a CD8 hinge, lowering the flexibility, can cause lower cytokine production of IFN-γ, IL-2, TNF-α and IL-6 in T cells (71). IgG hinges have also been associated with decreased CAR-T persistence *in vivo* due to their interaction with Fcγ domains therefore more origins for a variety of flexibilities and length hinges are being explored (72, 73).

The TMD anchors the CAR to the surface of the effector cell and facilitates the transfer of antigen binding signals inside the cell. Generally, CAR TMD’s are derived from type-I single spanning proteins such as CD3ζ, CD4, CD8α or CD28 (74). The TMD is the least researched component of CARs but has been shown to have effects on CAR expression level and stability. For example, CARs with TMD originating from CD8α and CD28 have been related to higher surface expression and increased CAR expression stability enhancing cytotoxic capacity (75). Though most CARs are designed as homodimers, CAR heterodimerization can also be mediated by certain TMDs such as CD3ζ TMD dimerization with endogenous TCR complexes and CD28 TMD dimerization with CD28 receptors, which can increase CAR signaling/sensitivity but potentially raise on-target off-tumor toxicity risks (76, 77).

Effective lymphocyte activation by a CAR relies on its ICDs to determine the highest cytotoxicity while maintaining safety. The first 3 generations of CAR were determined by the number of ICDs, with the original first-generation CAR intracellular domain consisting solely of a CD3ζ primary activation domain, second including one costimulatory domain and third including two costimulatory domains. The CD3ζ, derived from the TCR complex, is the crucial signaling component of the CAR providing signal 1 of activation into the cell. It contains immunoreceptor tyrosine-based activation motifs (ITAMs) that initiate downstream signaling cascades and cell activation, as a result of antigen binding to CAR. Costimulatory domains, such as CD28 and 4-1BB, provide signal 2 of activation, enhancing internal activation and improving persistence and determine the function of the CAR (78). Due to the more recent advent of iNKT cell-based therapies, 1st generation CARs have not been studied in iNKT cells, only second, third and fourth generation CARs are currently in preclinical models or clinical trials (**Figure 2**). Currently, CAR-iNKT cell engineering relies on conventional CAR signaling domains, due to the limited research on optimal domains specifically for iNKTs, with 4-1BB emerging as a key enhancer of cytotoxic activity (79, 80). Heczey et al., found that incorporating 4-1BB as a costimulatory domain shifted the cytokine secretion profile of iNKT cells toward a Th1-dominant response, unlike CD28 or CD3ζ (80). To support this, Poels et al. demonstrated that iNKT cells expressing 4-1BB-containing CARs specific for BCMA and CD38 exhibited a similar Th1-skewed polarization (79). Third generation CARs incorporated two costimulatory domains in series with the CD3ζ to enhance persistence, cytokine secretion, and cytotoxicity compared to second generation CARs (81). Though the results of 3rd generation CARs in iNKTs are limited, the combination of CD28 and 4-1BB has been promising *in vivo* with GD2 and CD19 CARs demonstrating prolonged survival and increased CAR-iNKT cell persistence and anti-tumor activity (80, 82). However, the use of other intracellular domains in NK cells and emerging in αβ T cells, such as DAP12, Dectin-1 and ICOS, may hold promise for enhancing innate-like killing, reducing exhaustion, and improving persistence (83–85).

Fourth-generation CARs also known as TRUCKs (T cells Redirected for Universal Cytokine Killing) incorporated additional DNA sequences encoding cytokines to be secreted upon activation of the cell, also known as cytokine armor. Armored CAR-iNKT cells can enhance persistence and expansion of iNKTs, boost anti-tumor activity and reprogram the TME to enhance tumor infiltration and reduce suppressive signals. IL-15 is a key enhancement for CAR-iNKTs, as evidenced by its inclusion in all CAR-iNKTs currently in clinical use, due to its well-established role in iNKT cell persistence and anti-tumor activity (86, 87). IL-21 armored B7-H3 CAR-iNKT cells demonstrate improved persistence and therapeutic efficacy in lung metastatic *in vivo* mouse models along with an increased percentage of CD62L-positive memory-like iNKT cells (88). A recent study also incorporated IL-12 armor into CAR-iNKT cells resulting in an increased polarization of iNKT cells to Th1 phenotype with memory features, prolonged persistence and antitumoral capability (89).

Recently, fifth-generation CARs have been developed by modifying second-generation CARs with a truncated IL-2 receptor β-chain domain featuring a binding site for the transcription factor STAT3. In this way, fifth-generation CARs, upon antigen recognition, can activate the JAK-STAT pathway, enhancing T cell expansion, survival and resistance to exhaustion, however, fifth-generation CARs are yet to be established in iNKT cells (90).

The unpredictable expansion and associated toxicities of CAR-T cells such as CRS or ICANS have driven the search for inducible elements, engineered mechanisms that allow precise control over CAR cell activation, function, or depletion in response to specific stimuli. While similar toxicities have not yet been observed in CAR-iNKT cells, their potential for extensive expansion and persistence and the utmost importance of patient safety necessitates the consideration of safety measures. Kill switches take advantage of intrinsic or extrinsic apoptotic pathways or antibody-dependent cell-mediated cytotoxicity (ADCC) and phagocytosis of remaining cell components, allowing for the rapid elimination of a therapeutic agent on demand in the instance of excessive expansion or toxicity (91). The most effective, clinically proven and widely used kill switch utilizes a modified Caspase-9 (iC9), which triggers apoptosis upon dimerization with FK506-binding proteins (FKBPs) (92). The dimerization of these proteins can be modulated with small molecule chemical inducer of dimerization (CID) agents such as AP1903 (Rimiducid), eliminating up to 90% of the peripheral blood CAR T cells within hours (93, 94). Drug-inducible activation switches, such as rapamycin-based dimerization systems, can also assemble CAR signaling domains only in the presence of a small molecule allowing regulation of activation (95). Altogether, CAR design continues to evolve to enhance safety and efficacy, with iNKT-specific optimization offering exciting potential for next-generation cell therapies.

## Engineered iNKT cells: combining innate sensing with CAR-targeted precision for superior anti-tumor immunity

CAR-engineered iNKT cells offer distinct advantages over conventional CAR-T therapies by integrating CAR-mediated precision targeting with innate immunomodulatory capabilities, tumor microenvironment reprogramming, and coordinated anti-tumor immunity. Preclinical and clinical evidence increasingly highlights their distinct advantages over conventional cellular therapies, particularly in their ability to infiltrate tumors, modulate myeloid populations, and establish durable immune responses.

A fundamental limitation of conventional CAR-T therapies is their inefficient trafficking to, and infiltration of, solid tumors. Heczey et al. demonstrated that iNKT cells engineered with CARs targeting GD2 ganglioside (CAR.GD2), a disialoganglioside highly expressed on neuroblastoma and other solid tumors (96), exhibited significantly enhanced tumor infiltration compared to similarly engineered conventional T cells in neuroblastoma xenograft models (80). This enhanced tumor-homing capacity, likely attributable to the distinct chemokine receptor profiles of iNKT cells, represents a critical advantage for targeting solid malignancies where limited infiltration often undermines therapeutic efficacy.

The most distinctive feature of engineered iNKT cells is their capacity to remodel immunosuppressive tumor microenvironments into immunostimulatory ones through coordinated immunomodulatory mechanisms. In preclinical mouse studies, autologous CAR-iNKT exhibit superior anti-tumor activity compared to conventional CAR-T cells across multiple syngeneic tumor models, including subcutaneous and lung metastatic B16-OVA-hCD19 tumors and D8-mB7-H3 ovarian cancer (97). Detailed mechanistic analyses using single-cell RNA sequencing, flow cytometry, and immunohistochemistry revealed that CAR-iNKT cells selectively depleted immunosuppressive myeloid populations through CD1d-dependent elimination of M2-like macrophages, while preserving CD1d-low M1-like macrophages with pro-inflammatory functions. This selective CD1d-dependent remodeling of the myeloid compartment was also observed with TCR-engineered iNKT cells, which controlled multiple tumors expressing cognate antigen more effectively than either non-transduced iNKT cells or CD8^+^ T cells engineered with the same TCR (98), further validating the translational potential of engineered iNKT platforms.

Beyond TME remodeling, CAR-iNKT promoted robust epitope spreading, eliciting broad immune responses against both engineered targets and endogenous tumor neoantigens, and enhanced the expansion of T cell memory, thereby establishing durable anti-tumor immunity that persists even after clearance of the transferred cells themselves (97, 99). While CAR-iNKT directly reduced tumor burden through CAR-mediated cytotoxicity in a syngeneic murine model of B cell CD19^+^ lymphoma, complete anti-tumor efficacy required dendritic cell-mediated cross-priming of host CD8^+^ T cells (99). Similarly, next-generation IL-15-armored, FAP-targeting CAR-iNKT therapy MiNK-215, designed to target the tumor stroma, further enhanced endogenous anti-tumor activity in human organoid models of treatment-resistant colorectal cancer liver metastases (22, 100, 101). MiNK-215, depleted FAP^+^ hepatic stellate cells and reduced CXCL12 expression to alleviate immune suppression, enhanced secretion of chemokines (CX3CL1, CXCL9, CCL3) to promote T cell trafficking, and boosted T cell activation and cytotoxicity through increased levels of effector molecules (sCD137, CD40L, IFN-γ, GZMB, sFASL) (100). This capacity to induce epitope spreading and activate broader host immunity represents a powerful strategy to overcome tumor heterogeneity and antigen escape mechanisms, which frequently limit the efficacy of conventional CAR approaches.

## iNKT cells as a universal platform for engineering safe, scalable, and effective off-the-shelf immunotherapy

**Figure 3:**
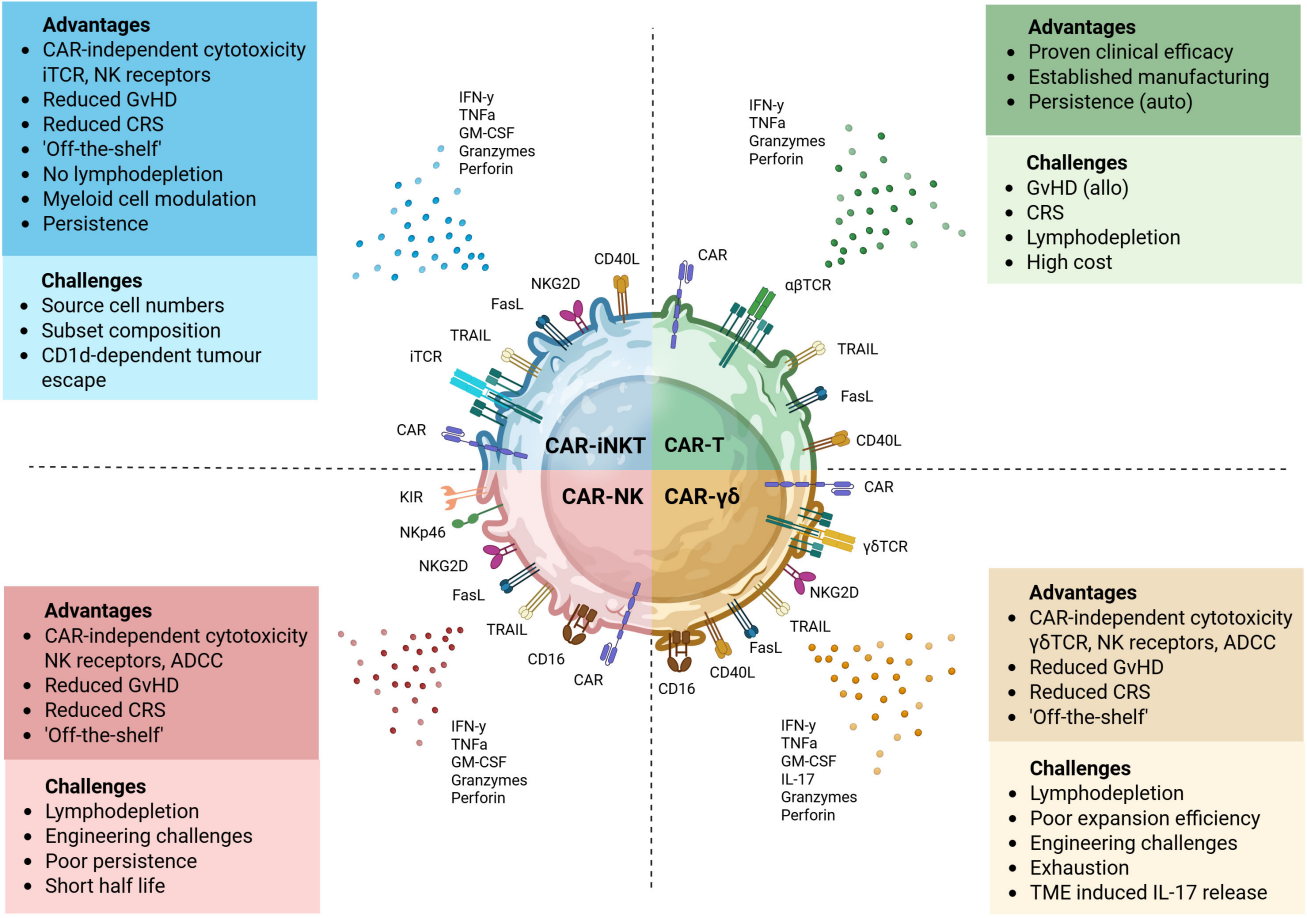
Key attributes of CAR-engineered lymphocyte platforms. Each CAR-engineered platform offers unique therapeutic benefits and presents specific limitations. Autologous CAR-T cells can provide potent and sustained tumor control, particularly in hematological malignancies. However, they are associated with significant safety concerns, including CRS, neurotoxicity and GvHD in allogeneic settings. CAR-iNKT, -NK and -γδ T cells offer simpler “off-the-shelf” manufacturing potential. Innate cytotoxicity of CAR-NK through activating receptors and cytokine secretion supports antigen-independent killing, but they may have reduced persistence and are difficult to engineer. CAR-iNKT cells combine adaptive and innate-like features, recognizing glycolipid antigens via their invariant TCR and mediating cytotoxicity through FasL, TRAIL, and NK receptors. They can also modulate the tumor microenvironment by promoting dendritic cell maturation and influencing macrophage phenotype. Their dual targeting capacity and lower alloreactivity make them promising. Ongoing studies are investigating how tumor-driven modulation of CD1d expression and lipid antigen presentation impairs iNKT recognition and function. In parallel, efforts aim to understand the functional differences between iNKT subsets and optimize their composition to improve the efficacy of CAR-iNKT therapies. CAR-γδT cells recognize a broad range of stress-induced or metabolic antigens independently of MHC and can mediate ADCC via Fc receptors like CD16. They can exhibit both pro- and anti-tumor activity and expansion protocols remain areas of active development. Image created in BioRender.

Promising safety profile makes CAR-iNKT cells ideal candidates for allogeneic ‘off-the-shelf’ development (**Figure 3**). In xenogeneic neuroblastoma models, GD2-targeting CAR-iNKT exhibited superior safety compared to conventional GD2-CAR-T cells, with no evidence of GvHD or multiorgan toxicity despite repeated administration, underscoring their preserved non-alloreactive properties post-engineering (80). More recently, PSCA CAR_sIL-15 iNKT and T cells showed similar antitumor efficacy in humanized mice, significantly suppressing tumor growth of metastatic pancreatic ductal adenocarcinoma (PDAC). However, only CAR-T cell–treated mice developed severe GvHD, splenomegaly, and elevated CRS-related cytokines. In contrast, CAR-iNKT treatment did not cause notable GvHD or CRS, indicating a safer therapeutic profile with comparable efficacy (102). This intrinsic non-alloreactivity represents a valuable advantage for developing allogeneic therapies, as conventional allogeneic T cell approaches can require genetic modification to overcome HLA barriers, including elimination of endogenous TCRs for GvHD and HLA molecules for host-versus-graft (103, 104). Such modifications introduce substantial manufacturing challenges. TCR removal impairs ex vivo expansion by preventing expression of the CD3 complex for T cell activation, signaling, and proliferation (105), while HLA deletion can render cells susceptible to NK cell-mediated elimination (106). Furthermore, the extensive gene editing required raises regulatory concerns regarding potential off-target effects and chromosomal abnormalities (107–109). iNKT cells circumvent these fundamental limitations, requiring no genetic manipulation to prevent GvHD alloreactivity and persistence has so far been encouraging without HLA manipulation (54–58).

Complementing their inherent allogeneic-related properties, the clinical advantages of iNKT extend further to patient preparation protocols. While conventional CAR-T therapies typically require intensive lymphodepletion regimens, which limit eligibility and could impact efficacy (110, 111) and is often associated with toxicities, including leukopenia, reactive myelopoiesis, and prolonged hospitalization (112, 113), iNKT cells demonstrate remarkable persistence and function in HLA-mismatched recipients without such intensive preconditioning (114). Clinical evaluation of agenT-797 confirmed this advantage, with cells persisting up to 6 months post-infusion without lymphodepletion or HLA matching (56).

Compared to alternative “off-the-shelf” approaches utilizing γδ T cells or NK cells, iNKT offer several practical advantages (**Figure 3**). NK cells, while potent effectors, are challenging to transduce due to innate immune sensors that trigger apoptosis following viral transduction, and they demonstrate poor persistence *in vivo* (115–117). Similarly, γδ T cells face limitations including poor expansion efficiency, difficulties in genetic engineering, and susceptibility to activation-induced cell death (118–121). Collectively, these intrinsic and engineered advantages position engineered iNKT cells as uniquely suited candidates for scalable, safe, and effective next-generation cellular immunotherapies, offering compelling alternatives to conventional CAR-T, NK, and γδ T-cell strategies.

## CAR-iNKT cells in action: a look at the clinical landscape

While extensive preclinical data supports the therapeutic potential of CAR-iNKT, their translation to clinical applications remains in early stages (21, 122–124). To date, only seven clinical trials evaluating CAR-iNKT have been initiated, with emerging data providing preliminary insights into their safety profile and therapeutic efficacy across both hematologic and solid malignancies (**Table 2**). Most use low dose IL-2 to expand CAR-iNKT cells with additional “armoring” with mainly IL-15 gene to date.

**Table 2:** Clinical trials of CAR-iNKT cells.

Trial ID	Start Date	iNKT cell product	Phase	Target	Indication	Response/Toxicity	ICDs	Armor	Reference
NCT03294954	January 2018	Autologous	1 (Recruiting)	GD2	R/R neuroblastoma	25% ORR, 3 PR, 4 SD (33.3%), 5 PD (41.7%) 1 case Grade 2 CRS (8.3%), Death of first patient on DL5 Post Second Dose: 1 PR → CR, 1 SD → PD, 1 PR → PD, 1 maintained PR	CD28, CD3ζ	IL-15	(21, 125)
NCT05487651	June 2020	Allogeneic	1 (Recruiting)	CD19	R/R B-NHL, ALL, CLL	3 CR (33.3%), 1 PR (11.1%) 1 case Grade 1 CRS (11.1%)	CD28, CD3ζ	IL-15	(122)
NCT03774654	June 2020	Allogeneic	1 (Recruiting)	CD19	R/R B cell malignancies	No results reported	CD28, CD3ζ	IL-15	
NCT04814004	March 2021	Allogeneic	1 (Status unknown)	CD19	ALL, B cell lymphoma	No results reported	–	IL-15	
NCT06182735	July 2023	Autologous	1 (Recruiting)	CD70	Renal cell carcinoma	50% ORR, 2 PR 1 case Grade 2 CRS (25%), 1 case Grade 2 ICANS (25%) Common adverse events: lymphodepletion-associated neutropenia, thrombocytopenia, and leukopenia.	–	–	(123, 124)
NCT06394622	April 2024	Autologous	1 (Recruiting)	CD70	Advanced Malignant Solid Tumours	No results reported	–	–	
NCT06728189	November 2024	Autologous	1 (Recruiting)	CD70	Advanced Malignant Solid Tumours	No results reported	–	–	

The table summarizes ongoing CAR-iNKT cell clinical trials and latest data, detailing cancer targets, CAR design, protein targets and generation, and iNKT cell origin (autologous or allogeneic). References are provided. ORR, overall response rate; PR, partial response; CR, complete response; SD, stable disease; PD, progressive disease; CRS, cytokine release syndrome; ICANS, immune effector cell-associated neurotoxicity syndrome; R/R, relapsed/refractory; B-NHL, B-cell non-Hodgkin lymphoma; ALL, acute lymphoblastic leukaemia; CLL, chronic lymphocytic leukaemia.

Three of the seven clinical trials target CD19-expressing hematologic malignancies. The ANCHOR trial (NCT03774654) and its follow-up ANCHOR2 (NCT05487651) are evaluating allogeneic CD19-targeting CAR-iNKTs in patients with relapsed or refractory B-cell malignancies, including B-cell non-Hodgkin lymphoma (NHL) and acute/chronic lymphocytic leukemia (ALL/CLL). These CAR-iNKTs incorporate CD28/CD3ζ intracellular signaling domains, IL-15 armoring for enhanced persistence, and express shRNAs targeting β2M and CD74 to downregulate HLA class I and II expression, respectively (122). In the phase 1 dose-escalation ANCHOR trial, nine patients (seven with NHL and two with ALL) received treatment across three dose levels. Encouraging efficacy signals were observed, particularly in the NHL cohort where three of seven patients achieved initial partial responses (PR), with two subsequently converting to complete responses (CR). Additionally, one of the two ALL patients achieved a CR with incomplete hematologic recovery. Importantly, CAR-iNKT demonstrated dose-dependent *in vivo* expansion, with cells detectable in peripheral blood one week post-infusion at higher dose levels. Although circulating CAR-iNKT were not detected beyond three hours in the lowest dose cohort, cells were identified in post-treatment tumor biopsies, confirming their ability to infiltrate target tissues even at low doses. The safety profile of this allogeneic product appears favorable, with only one patient experiencing mild CRS (122). A parallel approach is being evaluated in a separate trial (NCT04814004) using CD19-CAR-iNKTs armored with IL-15 in patients with acute lymphoblastic leukemia, B-cell lymphoma, or chronic lymphocytic leukemia, though results from this study have not yet been reported.

The application of CAR-iNKT to solid tumors has so far been explored in four clinical trials targeting distinct malignancies. The GINAKIT2 trial (NCT03294954) evaluated autologous GD2-CAR-iNKT incorporating CD28/CD3ζ intracellular domains and IL-15 secretion in pediatric patients with relapsed or refractory neuroblastoma. Initial results from twelve treated patients demonstrated a 25% overall response rate, including three partial responses, four cases of stable disease, and five instances of progressive disease (21). Among four patients who received a second infusion, one converted from PR to CR, one maintained PR, and two experienced disease progression. Importantly, this trial provided valuable insights into factors affecting therapeutic efficacy. Higher levels of CD62L^+^ CAR-iNKTs in the infused product correlated with improved patient outcomes, consistent with preclinical findings highlighting the importance of this memory-like subset for enhanced persistence and anti-tumor activity (21). However, the study faced a significant setback following a patient death, prompting temporary suspension by the FDA. Subsequent investigation attributed this adverse event to hyperleukocytosis resulting from manufacturing changes implemented to achieve higher cell doses. Analysis of the pre-infusion product revealed cell-autonomous expansion and outgrowth of NK cells (125).

More recently, autologous CD70-CAR-iNKTs (CGC729, NCT06182735) have shown promising activity in patients with relapsed and refractory metastatic renal cell carcinoma (mRCC). Preliminary results from four patients who received a single dose following lymphodepletion demonstrated an ORR of 50% (2/4) and a DCR of 75% (3/4) (123, 124). Notably, both patients with CD70-expressing RCC responded to therapy despite low antigen expression levels, suggesting that CAR-iNKT cells may effectively target tumors with limited antigen density; a significant advantage for solid tumor applications where heterogeneous and low antigen expression often limit conventional CAR-T efficacy. CGC729 exhibited favorable pharmacokinetics, with peak expansion between days 14 and 28 and persistence in circulation up to week 20. The durability of its biological activity was further evidenced by sustained reduction in peripheral CD70^+^ T cells through week 20. The treatment demonstrated a manageable safety profile with no dose-limiting toxicities. Most adverse events were attributable to lymphodepletion rather than the cellular product itself, primarily consisting of transient cytopenias. Only one patient experienced grade 2 CRS and grade 2 ICANS, both of which resolved rapidly with standard management (123, 124). Based on these encouraging results, CGC729 is now being evaluated in additional solid tumor indications (NCT06394622, NCT06728189), with data anticipated to further elucidate its potential across diverse malignancies.

As these clinical trials mature and additional studies commence, a more comprehensive understanding of the therapeutic potential CAR-iNKT therapy in humans will emerge. Nevertheless, These early findings already demonstrate the versatility, safety, and promising efficacy of CAR-iNKT therapies across diverse cancer indications, with significant potential to transform treatment paradigms where conventional cellular immunotherapies face significant limitations.

## Challenges in the development of CAR-iNKT cell therapy

Despite the significant therapeutic potential of CAR-iNKT cells, several critical challenges must be addressed to fully realize their clinical promise. These challenges span manufacturing hurdles, functional heterogeneity considerations, and tumor microenvironment-mediated resistance mechanisms.

A fundamental challenge in developing CAR-iNKT therapies arises from the inherent scarcity of iNKT cells, which constitute only 0.01–1% of human peripheral blood lymphocytes (29, 30), necessitating extensive ex vivo expansion to generate clinically relevant doses. This challenge is particularly pronounced in autologous settings, where cancer impacts endogenous iNKT (18, 19) and patients may have undergone multiple rounds of immunosuppressive treatments that further deplete iNKT populations and compromise their functionality. Despite these limitations, several successful expansion protocols have been developed, typically involving stimulating naïve iNKT cells with α-GalCer-pulsed antigen-presenting cells or anti-CD3 antibodies, combined with supportive cytokines such as IL-2, IL-7, IL-15, or IL-21 (126). The clinical feasibility of this approach was demonstrated in the GINAKIT2 trial conducted by Heczey and colleagues, where autologous GD2 CAR-iNKT expressing IL-15 were manufactured for children with neuroblastoma. Despite the low blood volume available and low starting frequency of iNKT cells (¾0.1% of peripheral blood lymphocytes) and patients’ histories of myeloablative and lymphodepleting chemotherapy, the expansion process achieved a median of more than 60% CAR+ cells and 10^8^ CAR+ iNKT per product, enabling repeat dosing for most patients (21, 127).

Alternative manufacturing approaches utilizing induced pluripotent stem cells (iPSCs) or hematopoietic stem or progenitor cells (HS/PC) as starting materials are being actively explored to circumvent the need for patient-derived iNKT cells (128–130). Yamada et al. successfully generated functional iNKT from iPSC, which demonstrated robust antitumor activity and enhanced NK cell responses in preclinical models (131). Additionally, universal CAR-engineered iNKT (UCAR-NKT) have been developed through ex vivo HSC differentiation, iNKT TCR engineering, HLA gene editing, producing large-scale, potent CAR-NKT populations with promising preclinical antitumor efficacy and safety profiles (130). Notably a single cord blood donor containing approximately 5 × 10^6^ CD34^+^ HSPC could potentially yield an estimated 10¹² mature allogeneic CAR^+^ iNKT, sufficient for ~1,000+ clinical doses (132). These advances establish a robust foundation for large-scale manufacturing of engineered iNKT as an “off-the-shelf” immunotherapy platform.

The functional heterogeneity of iNKT cells represents both an opportunity and a challenge for therapeutic development. As mentioned above, iNKT can be broadly classified into distinct subsets based on CD4 expression, with CD4^+^ cells demonstrating polyfunctional cytokine production (GM-CSF, TNF-α, IFN-γ, IL-4, IL-2 etc.) and CD4^-^ iNKT specializing in cytotoxicity and relatively more Th1 cytokines (133). The therapeutic relevance of these subsets was demonstrated in a xenograft model of Epstein-Barr virus-driven B-lymphoma, where CD4^+^ iNKT cells functioned in an adjuvant-like manner to enhance anti-tumor responses by antigen-specific T cells (134), while CD62L expression further distinguished memory-like iNKT, correlating with enhanced persistence, proliferation, and antitumor activity (135, 136). This preclinical observation was subsequently validated clinically, where the frequency of CD62L^+^ GD2-specific iNKT cells in pre-infusion products correlated with CAR-iNKT expansion in patients and was significantly higher in responders compared to non-responders (21). Interestingly, HS/PC-derived iNKT cells lack the CD4^+^ subpopulation commonly found in endogenous human iNKT, suggesting that peripheral blood-derived iNKT may serve as a more suitable starting material for generating functionally complete CAR-iNKT cell products (130, 132). Additional functionally distinct iNKT subsets include CD244^+^CXCR6^+^ iNKT with NK cell-like features, high IFN-γ and granzyme production, and enhanced anti-tumor activity (137). These cells belong exclusively to the CD4^-^ subset, further defining functional specialization among iNKT populations (137). Optimizing the composition and functional properties of these distinct iNKT subsets represents a key opportunity to enhance both therapeutic outcomes and predictability of CAR-iNKT therapies for specific clinical applications from cancer to immune/inflammatory, infectious and other diseases.

Lastly, while CAR-iNKT cells demonstrate remarkable capabilities to reshape the TME, they remain susceptible to various immunosuppressive mechanisms that could limit CAR-iNKT therapeutic efficacy. The lipid composition within tumors can significantly alter iNKT function, with many tumors accumulating immunoregulatory lipids that skew iNKT cells towards a tolerogenic phenotype (138, 139). In hepatocellular carcinoma, accumulated long-chain acylcarnitine induced premature iNKT cell senescence and impaired responsiveness to activating stimuli (140). CD1d-mediated interactions between iNKT cells and other immune populations also shape anti-tumor responses, sometimes in unexpected ways. In colorectal cancer (CRC), the iNKT-neutrophil axis paradoxically suppresses anti-tumor immunity, with tumor-infiltrating iNKT exhibiting a pro-tumorigenic phenotype characterized by GM-CSF and IL-17 secretion and expression of exhaustion markers (PD-1, TIGIT, TIM-3) (141). In contrast, iNKT in adjacent non-tumor tissue predominantly produced IFNγ (141), highlighting the critical impact of the local microenvironment on iNKT functional polarization. Metabolic challenges further compound these issues, as tumor-derived lactic acid suppresses PPARγ expression in iNKT, impairing IFNγ production and anti-tumor function (142, 143). Will allogeneic healthy donor iNKT restore the anti-tumor balance or be eventually impacted by the TME, perhaps therefore requiring further dosing. Elucidating these mechanisms presents both challenges and opportunities for the rational design of next-generation CAR-iNKT with broad anti-tumor efficacy and persistence. Promising strategies include engineering resistance to metabolic stress, incorporation of checkpoint blockade elements, and cytokine armoring to maintain functionality within a hostile TME (144, 145).

## The road ahead: opportunities and takeaways

While pre-clinical and early clinical data for CAR-iNKT demonstrate promising safety and efficacy, multiple opportunities exist to enhance their therapeutic potential and expand applications to difficult-to-treat cancers. Emerging strategies include combination therapies targeting complementary immune pathways, engineering approaches to enhance persistence and functionality, and innovative targeting strategies that address the broader TME beyond malignant cells themselves.

Strategic combinations of CAR-iNKT with checkpoint blockade and T-cell engagers (TCEs) represent a compelling opportunity to deepen clinical efficacy and overcome resistance mechanisms. Activation-induced programmed cell death protein 1 (PD-1) expression on iNKT has been documented in both mouse models (146, 147) and non-small cell lung cancer patients (148), suggesting that combining CAR-iNKT with checkpoint inhibitors could prevent exhaustion and enhance therapeutic outcomes. Pre-clinical data show that PD-1 blockade (nivolumab) enhanced CAR-iNKT proliferation, cytokine production (IL-2, TNF-α, IFN-γ, and granzyme B) and effectively reversed CAR-iNKT exhaustion upon tumor rechallenge, translating into deeper tumor control (149). Translating these findings to the clinic, a first-in-human study (NCT06251793) combining unmodified iNKT (agenT-797) with Fc-enhanced anti-CTLA-4 (botensilimab) and anti-PD-1 (balstilimab) antibodies demonstrated potent intratumoral immune activation and enhanced effector cell responses in PD-1–refractory gastrointestinal cancers (57), suggesting that combination with dual checkpoint inhibition may overcome resistance in PD-1-refractory solid tumors. Likewise, co-infusion of agenT-797 with CD3-based T cell Engagers (TCE, e.g., DLL3, HER2, Muc16 and CLDN18.2 bispecifics) broadens the therapeutic window in multiple tumor models by providing a pre-primed, co-stimulation-independent effector pool, enabling lower, and therefore safer TCE dosing (150). Optimization of sequencing and dosing will be essential to unlock the full clinical benefit of these combinations.

Long-term persistence of CAR effector cells correlates with durable clinical responses (151, 152), however tumor recurrence in preclinical models has been associated with limited CAR-iNKT cell persistence (153). While IL-15 armoring has been incorporated in current clinical trials (**Table 2**), emerging evidence suggests that alternative cytokine strategies may further enhance CAR-iNKT anti-tumor activity. In leukemia and neuroblastoma models, IL-12-expressing CAR-iNKT cells demonstrate superior anti-tumor efficacy compared to IL-15-expressing counterparts by promoting Th1 polarization and molecular reprogramming that enhances activation and proliferation while limiting exhaustion and generating long-lived memory iNKT cells (89). Similarly, IL-21 armoring enhances CAR-iNKT efficacy by increasing CD62L^+^ memory-like cell frequency and activating STAT3 signaling pathways critical for survival and effector function (88). In renal cancer xenograft models, these IL-21-armed cells achieved durable, recurrence-free tumor control without evidence of cytokine-related toxicity (88). These findings position IL-12 and IL-21 as promising armoring candidates to overcome exhaustion and enhance durability, while future approaches targeting metabolic fitness and resistance to tumor-derived immunosuppressive factors could further optimize CAR-iNKT efficacy across diverse malignancies.

Lastly, expanding targets beyond tumor cells could represent a transformative opportunity for CAR-iNKT cells to simultaneously address both malignant cells and the immunosuppressive components of the tumor microenvironment that enable immune evasion. This dual-targeting approach is exemplified by MiNK-215, an allogeneic IL-15-armored FAP-targeting CAR-iNKT therapy that demonstrated remarkable efficacy in models of treatment-resistant microsatellite-stable colorectal cancer liver metastases by eliminating FAP+ cancer-associated fibroblasts and promoting T cell infiltration and activation in tumors refractory to checkpoint blockade (22, 100, 101).

## Conclusion

iNKT therapy in general and CAR-iNKT cell therapy in particular represent transformative approaches in cellular immunotherapy. The latter combines the precision targeting of chimeric antigen receptors with the unique immunomodulatory properties of iNKT. Inherent advantages of iNKT cells include their remarkable proliferative capacity without exhaustion, capability to be produced ultra-pure with mono- iTCR specificity, iTCR-specific control *in vivo*, non-alloreactivity, enhanced tumor infiltration capacity, cytotoxic potential, and ability to reshape the tumor microenvironment. These properties address many limitations of conventional CAR-T and other CAR+ cell approaches. Early clinical trials have demonstrated encouraging safety and efficacy across both hematologic and solid malignancies, while preclinical studies continue to reveal innovative strategies to enhance persistence, functionality, and therapeutic range. As research progresses, CAR-iNKT platforms are likely to expand beyond oncology into inflammatory, autoimmune, and age-related conditions, leveraging their versatile immunomodulatory properties. By capitalizing on the unique advantages of iNKT cells, CAR-iNKT therapy is poised to emerge as a next-generation approach in cellular immunotherapy with broad applications across human disease.
